# Retracted: Novel Hybrid Gels Made of High and Low Molecular Weight Hyaluronic Acid Induce Proliferation and Reduce Inflammation in an Osteoarthritis *In Vitro* Model Based on Human Synoviocytes and Chondrocytes

**DOI:** 10.1155/2022/9873639

**Published:** 2022-02-22

**Authors:** BioMed Research International

*BioMed Research International* has retracted the article titled “Novel Hybrid Gels Made of High and Low Molecular Weight Hyaluronic Acid Induce Proliferation and Reduce Inflammation in an Osteoarthritis *In Vitro* Model Based on Human Synoviocytes and Chondrocytes” [1], due to concerns with the reliability of the data. In January 2020, the journal was made aware of concerns with figure duplications as noted on PubPeer [2]. The journal proceeded to investigate these concerns, however, the authors separately contacted the editorial office to request a corrigendum, which was published without consideration of the ongoing investigation [3].

The figure duplications identified are as follows:

- In Figure 6(a), the 24 h and 48 h panels for HCC are overlapping
- In Figure 6(a), the 72 h and 96 h panels for H-HA are overlapping

Following the conclusion of our investigation, which has also resulted in the retraction of another article [4], the article is being retracted from the journal with the agreement of the editorial board.

The authors do not agree to the retraction and the notice.
